# Translation, Cultural Adaptation of the Portuguese Provider Attitudes toward Cardiac Rehabilitation and Referral (PACRR-P) Scale and Assessment of Its’ Measurement Properties

**DOI:** 10.3390/healthcare12191954

**Published:** 2024-09-30

**Authors:** Mayara Moura Alves da Cruz, Luiz Carlos Marques Vanderlei, Carolina Takahashi, Maria Julia Lopez Laurino, Murilo Reis Alves da Cruz, Sherry L. Grace, Gabriela L. M. Ghisi

**Affiliations:** 1 School of Technology and Sciences, São Paulo State University (UNESP), Presidente Prudente, São Paulo 19060-900, Brazil; 2 University Center of Adamantina (UNIFAI), Adamantina 17800-000, Brazil; 3 Educational Foundation of the Municipality of Assis (FEMA), Assis 19807-130, Brazil; 4 Faculty of Health, York University, Toronto, ON M3J 1P3, Canada; 5 KITE Research Institute, University Health Network, University of Toronto, Toronto, ON M5G 2C4, Canada

**Keywords:** attitudes, cardiac rehabilitation, health knowledge, physician’s practice patterns, practice, questionnaires

## Abstract

Background/Objectives: Access to cardiac rehabilitation (CR) is contingent upon physician referrals, yet these are often inadequate, particularly in low-resource settings. This multi-method study aimed to translate, culturally adapt, and validate the Portuguese version of the Provider Attitudes toward CR and Referral (PACRR-P) scale, as well as to identify key factors influencing CR referral in a Latin American context for the first time. Methods: The PACRR was translated into Brazilian Portuguese through a rigorous process involving independent translation, back-translation, and expert panel review to ensure face, content, and cross-cultural validity. A total of 44 Brazilian physicians completed the questionnaire, allowing for an assessment of internal consistency, criterion validity, and convergent validity. Results: The findings confirmed the face, content, and cultural validity of the 20 translated items, with a mean item clarity rating of 4.8/5. The final version included 17 of the original 19 PACRR-P items, with a Cronbach’s alpha of 0.73. Referral rates were significantly associated with over one-third of the PACRR-P items, preliminarily supporting the scale’s criterion validity, while correlations with the ReCaRe scores further supported its convergent validity. The most prominent barriers to referral were a lack of familiarity with CR site locations, absence of a standard referral form, and lack of automatic referral processes. Conclusions: The PACRR’s validity and reliability among Portuguese-speaking providers are preliminarily supported.

## 1. Introduction

Cardiovascular diseases (CVDs) are among the leading causes of morbidity worldwide [1], including in Brazil [2,3]. Moreover, the number of individuals with ischemic heart diseases is growing exponentially; in Brazil, it has more than doubled in the past 20 years, reaching 4 million in 2019 [2]. Cardiac rehabilitation (CR) is an outpatient model of secondary preventive care to mitigate this burden. CR participation reduces CVD morbidity and mortality by 20% [4]. Major cardiac practice guidelines [5] as well as the Brazilian Guidelines for CR [6] highlight the importance of this intervention in the care of people living with CVDs [5]. CR is, however, under-utilized, particularly in lower-resource settings [7,8,9].

Health systems, referring providers, programs, and patient level barriers are at play [10,11]. Given patient’s CR access is dependent upon provider referral, provider-related factors are key. Indeed, studies evaluating patient-level barriers to CR participation—including in Brazil—have identified provider-related factors, such as physicians not encouraging them [12,13,14]. A systematic review of physician factors associated with CR referral identified 17 studies—none had been undertaken in low-resource settings such as South America [15]. There have been some qualitative studies on provider-related barriers to CR referral in low-resource settings [16,17], but to our knowledge, few used a validated quantitative measure [18].

The Provider Attitudes toward Cardiac Rehabilitation and Referral (PACRR) scale was developed to enable reliable and valid assessment of these [19], given how instrumental referral and encouragement are to patient access to these life-saving programs [20]. This scale was developed in a high-resource setting and has only been administered once in a low-resource setting (Western Pacific) [21]. It is likely that some adaptation of the PACRR is needed for low-resource settings given differences in the CR context. For instance, the availability of programs to which providers can refer is limited [7]; accordingly, automatic referral processes are not used to our knowledge [22,23]. It is also a likely consequence that many physicians are not familiar with CR program locations and benefits [24]. Moreover, CR programs are more often privately run [25], and patients have more barriers to attending (e.g., transportation and program costs) [26].

For these reasons, a better understanding of physician attitudes in these contexts is needed to support change. Therefore, the objectives of the present study were to: (1a) translate and culturally adapt a Brazilian Portuguese version of the PACRR Scale (PACRR-P), (b) establish its measurement properties, and (2) characterize the main factors impacting physician CR referral in a low-resource Latin American context for the first time.

## 2. Materials and Methods

### 2.1. Design and Procedures

**Figure 1:**
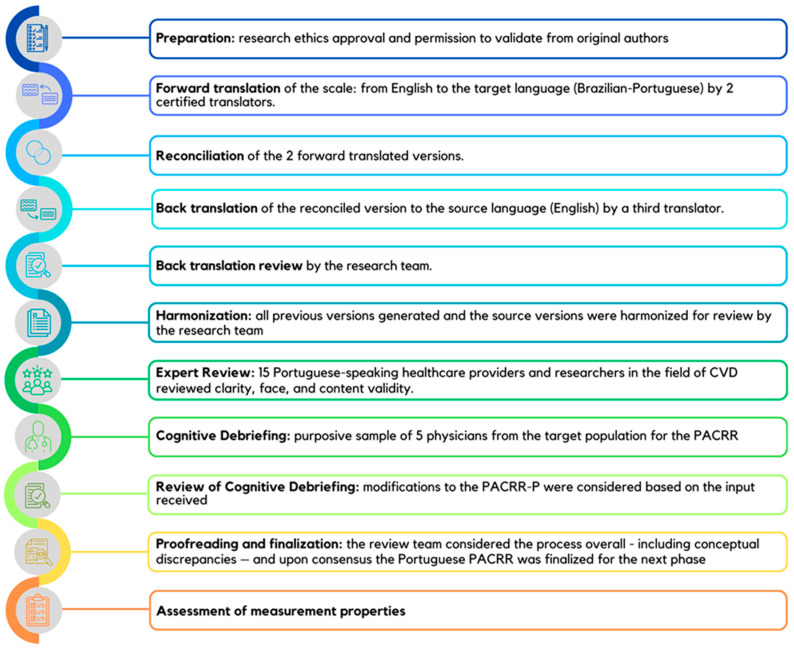
Processes to develop the PACRR-P. CVD: cardiovascular disease; PACRR-P: Provider Attitudes toward Cardiac Rehabilitation and Referral—Portuguese Version.

This was a multi-method study with 2 phases: (1) translation and cultural adaptation following best practices [27], followed by (2) a cross-sectional survey of physicians to assess the measurement properties and establish the top factors impacting referral (Figure 1). For the latter, reliability (internal) as well as several forms of validity were assessed: face, content, cross-cultural, convergent and criterion. Data were collected from November 2022 to March 2023.

### 2.2. Materials

The PACRR scale was developed by Ghisi and Grace (2019) to assess attitudes, beliefs and other factors that impact providers’ CR referral practices [19]. It was developed following an extensive literature review and input from health care professionals with expertise in CR. The PACRR comprises 19 items scored on a Likert-type scale, with response options ranging from 1 = strongly disagree to 5 = strongly agree. Five items are reverse scored to mitigate acquiescence bias. Higher scores reflect more positive attitudes toward CR and referral. A final open-ended item asks providers to list the most important factors that influence their decision to refer a patient to CR. The PACRR comprises four subscales, namely: referral norms, preference to manage patients independently of CR, perceptions of program quality, and referral processes. Internal reliability and validity of the scale were established [18]. It has been administered in a low-resource setting [19]. Although many providers globally train and/or are fluent in English, a Simplified Chinese translation has been validated [28].

### 2.3. Translation and Cultural Adaptation

The Professional Society for Health Economic and Outcomes Research (ISPOR)’s 10 steps were followed, as per Figure 1 [27]. With regard to the first step (preparation), study approval was secured from the Sao Paulo State University’s Research Ethics Board (CAAE:60124622.5.0000.5402). Also, permission to validate a Portuguese version of the PACRR was obtained, and authors of the original PACRR developmental study were invited to be part of this project. Written informed consent was obtained from all participants for inclusion in the study.

Forward translation of the scale from English to the target language (Portuguese) was performed by two certified translators fluent in both languages independently. Translators were first provided a clear explanation of the basic concepts related to the scale, with the intention that the translations would capture the conceptual meaning of the questions rather than being a literal translation. In order to resolve discrepancies between the forward translations, they were then reconciled into a single forward translation, which was then back translated to the source language (English) by a third certified translator. This back-translated version was then reviewed by the research team. As an additional quality control step to ensure that all discrepancies were considered, all previously generated and the source versions were harmonized for review by the research team.

Next, a review committee comprised of 15 Portuguese-speaking healthcare providers and researchers in the field of CVD (4 cardiologists, 1 general practitioner, 1 nurse, and 9 physical therapists) was asked to provide input on the translation. This included rating the clarity of each item (Likert-type scale ranging from 1 = very unclear to 5 = very clear). They were also asked to comment in an open-ended manner on face as well as content validity (including any items that should be added or deleted) and the cross-cultural relevance of the items. Modifications were considered based on the initial input, and the experts were asked to review the revised survey similarly, including rating of clarity; this was to continue until ratings were satisfactory and no further modifications to the scale were needed. 

Then, cognitive debriefing of the scale was performed with a purposive sample (i.e., working in the public and/or private sector; generalists, specialists, and subspecialists; junior and senior) of physicians from the target population for the PACRR (i.e., treats patients indicated for CR [e.g., coronary heart disease +/− revascularization, heart failure] and that are eligible to refer patients to CR) [24]. In line with best practices recommended by ISPOR [28], 5 participants were sought. They were provided the scale and asked to rate the clarity of each item as above and to provide open-ended input on face, content, as well as cross-cultural validity online. Modifications to the PACRR-P were considered based on the input received. 

Finally, the review team considered the process overall, including conceptual discrepancies. Upon consensus, the Portuguese version of the PACRR was ready for assessment of its measurement properties.

### 2.4. Assessment of Measurement Properties—Participants

The only healthcare professional type that can refer patients to CR in Brazil are physicians, and hence a convenience sample of such physicians was sought. Portuguese-speaking physicians who treat patients indicated for CR (e.g., cardiologists, general practitioners) working in Brazil were eligible to participate. We sought 100 participants for assessment of the measurement properties (i.e., 5 participants per item) [29]. Respondents who answered less than 80% of the PACRR items were excluded. 

### 2.5. Assessment of Measurement Properties—Procedure

Physicians were recruited through social media accounts from the UNESP’s School of Technology and Sciences Research Laboratory and by direct emails to eligible physicians at universities located in the state of São Paulo, Brazil (where the density of CR is highest, such that there are programs to which physicians can refer their patients) [7]. Recruitment occurred between October 2022 and February 2023. To optimize the survey response rate, we incorporated components of Dillman’s Tailored Design Method [30]. Informed consent was obtained online prior to initiating the survey. The online questionnaire was administered using Research Electronic Data Capture (REDCap). 

### 2.6. Assessment of Measurement Properties—Measures

Participant’s sociodemographic and occupational characteristics were assessed via self-report. Respondents were also asked about referral practices to investigate criterion validity. Items queried whether they generally refer to CR (yes/no). They were also asked to estimate the percentage of indicated patients they refer to CR per month.

To assess convergent validity, the Recommending Cardiac Rehabilitation Scale (ReCaRe) was also administered [31]. It is comprised of 17 items assessing health professionals’ attitudes, values, and beliefs regarding CR referral. While not synonymous with the PACRR given some differences in focus (e.g., the perceived severity and susceptibility subscale differs from the PACRR), we are not aware of any other validated scales in this area [19]. Items are rated on a Likert-type scale ranging from 1 = strongly disagree to 5 = strongly agree. Scores range from 17–85; higher scores denote items that have a greater influence on decision-making when recommending CR.

### 2.7. Assessment of Measurement Properties—Data Analysis

The Statistical Package for Social Sciences version 28 (SPSS Inc., Chicago, IL, USA) was used for the assessment of measurement properties. The level of significance for all tests was set at 0.05. 

A mean total PACRR score was computed. Subscale scores were also computed based on the original version [19]. Internal consistency was determined by calculating Cronbach’s *α*; a *value* > 0.70 was considered acceptable [32,33]. Finally, to assess convergent validity—given that the data for both the PACRR and RECARE are normally distributed—differences in the PACRR item scores by CR referral practices as well as subscale scores by the ReCaRe total scores were tested using Student’s independent samples *t*-tests and Pearson’s correlations, respectively.

## 3. Results

### 3.1. Translation and Cultural Adaptation

**Figure 2:**
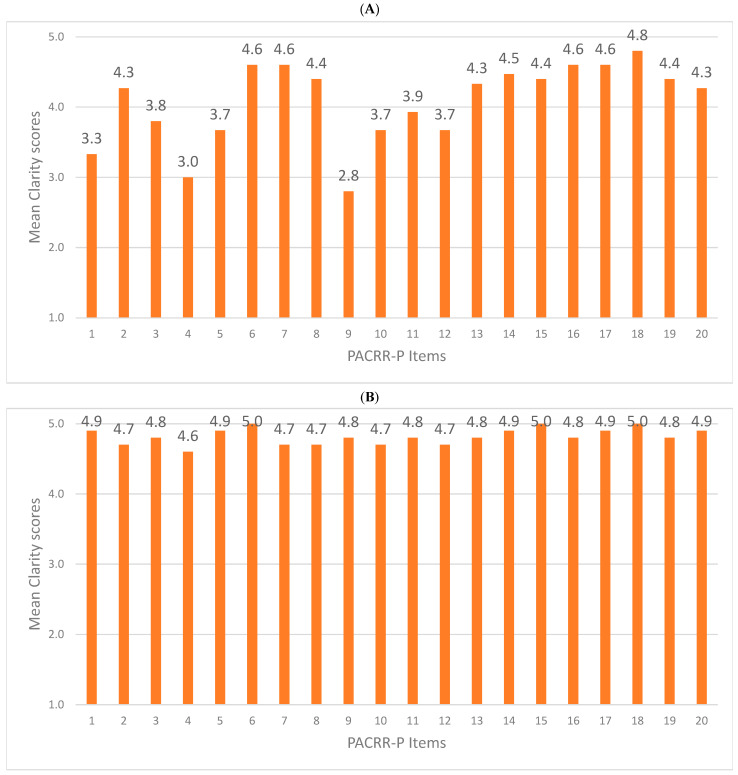
Mean PACRR-P item clarity ratings from round 1 (**A**) and round 2 (**B**), N = 15. Clarity scores ranged from 1 = very unclear to 5 = very clear.

Following translations, reconciliation, and harmonization of the PACRR to Portuguese as per Figure 1, the review committee deemed all 19 items in the original PACRR version applicable to the Brazilian context and had none to add. Clarity ratings of each item in round 1 are shown in Figure 2A and in round 2 in Figure 2B. As displayed, the clarity ratings ranged from 2.8–4.8/5 (mean 4.1 ± 0.6). Based on the qualitative input as well, minor wording changes were made to increase the clarity of items 1–16 and 19. For the second round of ratings from the review committee, the clarity ratings ranged from 4.7–5.0/5 (mean 4.8 ± 0.1); the scale was deemed ready for the next stage of testing.

Based on the cognitive debriefing of the scale performed with five physicians, the clarity of items ranged from 4.6 to 5.0/5 (mean 4.8 ± 0.1). None of the respondents selected the “not applicable” option for any item. Therefore, no further changes were made. 

### 3.2. Assessment of Measurement Properties of the PACRR-P

Other than the social media posting, the email invitation to complete the online survey was sent to 590 physicians (330 of them cardiologists). Ultimately, 44 (7.5%) completed ≥80% of the PACRR-P items (n = 33 excluded due to missing). Their characteristics are shown in Table 1; responses were from the two Brazilian regions with the most CR programs available in the country [7]. 

Table 2 displays the PACRR-P scores by item and subscale. It also displays the proportion of respondents that rated each item inapplicable, which ranged from 0–14% (mean = 5.0%). This further supports the content validity of the PACRR-P. The Cronbach’s alpha for the overall scale was 0.71, supporting the internal consistency of the scale.

Half of the physicians reported generally referring their patients to CR (Table 2), with 27.8 ± 30.5% of their patients referred. In regards to criterion validity, referral was significantly associated with PACRR-P items 3, 7, 8 and 10 (*p* < 0.05; trend for items 6, 9, 15; Table 2); no other significant associations were observed. Finally, the “perception of program quality” PACRR-P subscale was significantly correlated with the total ReCaRe scores (r = 0.31, *p* < 0.05), supporting convergent validity.

### 3.3. Brazilian Physician’s Attitudes toward Cardiac Rehabilitation and Referral 

Items with the highest scores (i.e., the most important factors) were as follows: not being familiar with CR sites outside their geographic area, not having a standard referral form for CR, not having an allied health professional fill out referral forms on their behalf, lack of standard institutional processes to support referral (e.g., automatic referral), and non-referral being normative. Items with the lowest scores (i.e., the least important factors) were related to being skeptical about the benefits of CR and having had a bad experience with a CR program.

In regard to the open-ended responses to question 20 about “the most important factors that affect your referral of patients to CR”, 34 valid responses were coded. Most respondents reported not knowing about CR programs in their area or outside their geographic area (n = 24), which aligns with the high rated PACRR-P items, supporting the content and face validity of the scale. 

Other main factors reported to affect CR referral were cost, access and availability of CR programs, followed by perceptions of patient motivation. Based on this, several changes were made to finalize the PACRR-P. Specifically, item 17 was revised to also include cost concerns, item 15 was revised to reflect amotivation for any patients (not just females), and item 19, which was considered least applicable, was revised to “Patients have too many barriers to attend CR, so there is no point in referring them” (Supplementary Materials).

## 4. Discussion

Despite well-established benefits, physician referral to CR is low in Brazil [6]. With the use of PACRR-P, factors that impede referral could be understood, and corresponding strategies to mitigate them can be developed and implemented, so that ultimately more patients can access CR. Following best practices, this study has rigorously translated and cross-culturally adapted a Portuguese version of the PACRR scale. Through this process, all 19 items of the scale were retained, with revisions made to 18 items to improve their clarity or ensure that all major factors were assessed. Face, content, and cross-cultural validity were supported. Measurement properties were tested; internal consistency as well as convergent and criterion validity were also preliminarily supported. 

With regard to the latter point, the criterion validity of the PACRR was not as robust as was evidenced in three North American cohorts [19]. This could be due to the smaller sample size in this study leading to a lack of power, but also may be due to the low-resource context. With a dearth of programs to which physicians can refer, attitudes and perceptions as well as referral processes are less relevant to CR referral practices. While some revisions were made to the PACRR (including to the English version; see: https://sgrace.info.yorku.ca/cr-barriers-scale/pacrr/ (accessed on 1 September 2024) for the PACRR-R) to ensure its applicability to low-resource settings, application of the PACRR in a given region should likely be reserved until structural issues such as program availability are addressed (i.e., applicable for use in settings with a higher density of CR than Brazil) [34].

The most strongly-endorsed attitudes impeding referral were not being familiar with the location of CR sites, not having a standard CR referral form and lack of automatic referral processes.

Total PACRR-P scores were lower than the mean in the original English validation (3.7 ± 0.4) [19], indicative of less positive attitudes toward CR and referral in the current low-resource sample. Otherwise, results from this study are generally consistent with other qualitative and quantitative studies assessing factors affecting physician CR referral, in low and high-resource settings alike. In our review of physician factors affecting referral [15], geographic issues, perceptions of patient motivation, patient clinical status and insurance coverage/cost were paramount. All of these were raised herein. In the only other administration of the PACRR in a low-resource setting (Philippines) [21], again, the main factors were concordant with the current findings, namely: costs, geographic issues/program accessibility, quality of the CR program, patient preferences/motivation, as well as financial incentives for the referral, lack of standard referral forms, and preference to manage secondary prevention of patients independent of CR. As described above, indeed, some revisions were made to the PACRR to render it more applicable to low-resource contexts through this study. 

With regard to implications, the PACRR-P can be administered to understand low CR referral rates and create strategies to mitigate them. Clinical associations, policymakers, and institutions could administer this tool to their cardiac physicians and work with them to overcome referral barriers. There is great need to augment funded CR capacity so that physicians have a place to refer their patients (and it can become normative), close to their home [7,35]. With more programs, automatic referral could be instituted where referral processes are identified by physicians as barriers. And acute cardiac care providers need to be educated on the benefits of CR, which patients should be referred (including education about valid and invalid clinical exclusions) [36] and how to refer them, where programs are located and how to encourage patients to attend. Such a course is freely available online in Portuguese (https://globalcardiacrehab.com/CR-Utilization (accessed on 1 September 2024), among other languages. This course has been shown to increase CR knowledge, referral self-efficacy, and patient encouragement among providers [37,38]. It could be broadly disseminated to non-referring physicians in an effort to increase CR utilization. 

Caution is warranted when interpreting these results. Generalizability is limited due to the small sample size (particularly for the validation of the measurement properties). This is due to the poor response rate to online surveys, particularly by physicians [39]. This also raises the possibility of selection bias. To optimize the survey response rate, we incorporated components of Dillman’s Tailored Design Method [30], including multiple contacts, personalized mailings and a short questionnaire. In a review of physician response to surveys [40], the demographic characteristics of late respondents (considered a proxy for non-respondents) were similar to the characteristics of respondents to the first mailing. Moreover, physicians as a group are more homogeneous than the general population with regard to knowledge, training, attitudes and behavior, suggesting that non-response bias may not be as crucial in physician surveys as with the general population [39].

Generalizability to other areas of Brazil, which are not as advantaged, and to other countries where Portuguese is a common language, is also unknown, particularly to jurisdictions with different healthcare systems, and to high-resource settings. For example, in Brazil, CR programs are often less comprehensive and of longer duration than other countries [7,41]. This concern is tempered, however, by the fact that the original scale was applicable to another health systems and was validated in a high-income country, and only minor wording changes were made in the Portuguese translation and adaptation process. 

Relatedly, future studies should focus on confirming the measurement properties of the finalized scale in a larger and more heterogeneous sample, as well as evaluating others (i.e., test-retest reliability, construct validity, responsiveness, interpretability, and hypothesis-testing) [42]. In addition, criterion validity was assessed herein through physician reports of referral, but it should be tested against verified referrals. 

Moreover, an assessment of factor structure is now warranted given some changes were made to the items, and the low-resource context. Another validated translation is also from a low-resource context, namely China (PACRR-C [45). They removed item 4 (reimbursement for referral) as it was not applicable in the local context. All other items were retained, with many wording revisions to optimize cultural validity, as with the Portuguese translation. Their PACRR-C factor analysis and structural equation model supported the four-factor structure of the scale [43]. This lends credence that the factor structure of the translation may hold, but again, future research is needed.

One final important area of future research arises from this work. Ways to overcome identified factors hampering provider referral such as those identified above should be tested in terms of how they impact referring physician attitudes.

## 5. Conclusions

Through this study, a Portuguese version of the PACRR-R applicable for use in low-resource settings was developed, and while more research is needed, its measurement properties were preliminarily supported as favorable. The main barriers to physician CR referral were identified as a lack of the following: familiarity with the location of CR sites, standardizing CR referral forms, and implementing automatic referral processes. These barriers are likely surmountable with provider education, CR program acceptance of discharge summaries or coordination of referral paperwork, and referral process improvements. 

## Figures and Tables

**Table 1:** Characteristics of respondents for the assessment of measurement properties (N = 44).

	n (%)/Mean ± SD
Age	45.1 ± 11.7
Sex	
Male	22 (50.0)
Female	22 (50.0)
Brazilian region of practice	
Southeast	39 (88.6)
South	4 (9.1)
Medical specialty	
Cardiology	18 (40.9)
Family Practice	12 (27.3)
Internal Medicine	8 (18.2)
Cardiac Surgery	3 (6.8)
Geriatrics	3 (6.8)
Years of practice	19.1 ± 13.0
Institutional funding	
Public	31 (70.5)
Private	13 (29.5)

CR: cardiac rehabilitation; PACRR-P: Portuguese Version of the Provider Attitudes toward Cardiac Rehabilitation and Referral; SD: standard deviation. Valid responses reported.

**Table 2:** PACRR-P mean scores, overall and by CR referral practices.

Item	Total (N = 44)	N/A (n, %)	Generally Refer to CR		
			Yes (n = 23; 52%)	No (n = 21; 48%)	P †
1. Clinical practice guidelines promote referral to CR *	1.8 ± 1.1	0 (0.0)	1.9 ± 1.4	1.6 ± 1.8	0.46
2. My colleagues generally refer patients to CR *	3.3 ± 1.3	1 (2.3)	3.3 ± 1.4	3.3 ± 1.2	0.95
3. My department/practice generally refers all eligible patients to CR as a standard of care *	3.3 ± 1.4	1 (2.3)	2.7 ± 1.5	3.9 ± 1.0	0.004
4. Reimbursement policies are a financial disincentive to CR referral	3.1 ± 1.3	2 (4.5)	3.1 ± 1.5	3.0 ± 1.2	0.84
5. Follow-up care, including referral, is handled by another healthcare professional	2.6 ± 1.3	2 (4.5)	2.6 ± 1.4	2.5 ± 1.3	0.83
6. I generally intend to refer patients to CR *	1.8 ± 1.1	1 (2.3)	1.6 ± 1.0	2.1 ± 1.1	0.13
7. I am not familiar with the CR programs in my area	3.0 ± 1.5	0 (0.0)	2.3 ± 1.3	3.8 ± 1.3	<0.001
8. I am not familiar with any CR sites outside my geographic area	3.7 ± 1.3	0 (0.0)	3.3 ± 1.5	4.2 ± 1.0	0.02
9. There is no standard referral form for CR, making it more effort to refer to sites closest to patients’ homes	3.7 ± 1.3	2 (4.5)	3.4 ± 1.4	4.1 ± 1.2	0.13
10. An allied health professional fills out referral forms on my behalf *	3.3 ± 1.4	3 (6.8)	3.4 ± 1.3	2.9 ± 1.5	<0.05
11. It is inconvenient to make a referral to CR	2.3 ± 1.3	3 (6.8)	2.2 ± 1.2	2.5 ± 1.3	0.37
12. I prefer to manage my patients’ secondary prevention myself	2.0 ± 1.2	3 (6.8)	2.1 ± 1.4	1.8 ± 1.0	0.32
13. I have patient education materials in my office that are sufficient for promoting behavioral change	1.9 ± 1.1	3 (6.8)	2.1 ± 1.2	1.7 ± 1.0	0.22
14. I can prescribe an exercise regimen for my patients myself	2.5 ± 1.2	1 (2.3)	2.4 ± 1.4	2.6 ± 1.0	0.63
15. Female cardiac patients generally do not like to exercise	2.1 ± 1.1	2 (4.5)	1.8 ± 1.0	2.4 ± 1.2	0.07
16. I am skeptical about the benefits of CR	1.3 ± 0.8	1 (2.3)	1.3 ± 0.9	1.2 ± 0.6	0.63
17. The available CR program is of poor quality	2.0 ± 1.2	4 (9.1)	1.9 ± 1.3	2.1 ± 1.1	0.44
18. I have had a bad experience with a CR program	1.5 ± 1.1	6 (13.6)	1.5 ± 1.2	1.4 ± 1.0	0.88
19. The CR program does not provide me with patient discharge summaries	2.3 ± 1.4	6 (13.6)	2.4 ± 1.5	2.1 ± 1.4	0.50
Subscale 1: Referral norms	2.6 ± 0.5	-	-	-	-
Subscale 2: Preference to manage patients independently of CR	2.0 ± 0.6	-	-	-	-
Subscale 3: Referral processes	3.5 ± 1.1	-	-	-	-
Subscale 4: Perceptions of program quality	2.6 ± 0.9	-	-	-	-
Total PACRR-P	2.5 ± 0.5	-	2.3 ± 0.5	2.5 ± 0.5	0.19

CR: cardiac rehabilitation; PACRR-P: Portuguese Version of the Provider Attitudes toward Cardiac Rehabilitation and Referral; N/A: not applicable; Mean scores ± standard deviation shown, unless otherwise indicated (1 = strongly disagree to 5 = strongly agree). * reverse-scored; † for independent samples *t*-tests.

## Data Availability

The data are available upon request. Please contact the corresponding author.

## Supplementary Materials

The following supporting information can be downloaded at: https://www.mdpi.com/article/10.3390/healthcare12191954/s1, Supplementary File: The Portuguese Version of the Provider Attitudes Toward Cardiac Rehabilitation and Referral Scale—Revised Atitudes Médicas em Relação à Reabilitação Cardíaca e Encaminhamento para os Programas (PACRR-P-R).
