# Supplementary material for: Translation, Cultural Adaptation of the Portuguese Provider Attitudes toward Cardiac Rehabilitation and Referral (PACRR-P) Scale and Assessment of Its’ Measurement Properties
Source: Healthcare (Basel). 2024 Sep 30;12(19):1954. doi: 10.3390/healthcare12191954 (PMC11477319; doi:10.3390/healthcare12191954)
Supplement: Supplementary file 1 [file healthcare-12-01954-s001.zip › healthcare-3216213-supplementary.pdf]

The Portuguese Version of the Provider Attitudes Toward Cardiac Rehabilitation & Referral Scale - Revised
Atitudes Médicas em Relação à Reabilitação Cardíaca e Encaminhamento para os Programas (PACRR-P-R)

**Por favor, indique o quanto você concorda ou discorda com as seguintes afirmações sobre reabilitação cardíaca (RC):**<br>***Please indicate how much you agree or disagree with the following statements about cardiac rehabilitation (CR):***	**Concordo totalmente**	**Concordo**	**Neutro**	**Discordo**	**Discordo totalmente**	**N/A**
1. Diretrizes de prática clínica (documentos informativos que incluem recomendações dirigidas a otimizar o cuidado prestado ao paciente) orientam o encaminhamento dos pacientes para RC.*<br>*1. Clinical practice guidelines (informative documents that include recommendations aimed at optimizing patient care) guide the referral of patients to CR.**	☐	☐	☐	☐	☐	☐
2. Outros profissionais da área da saúde que conheço geralmente encaminham pacientes para RC.*<br>*2. Other healthcare professionals I know usually refer patients to CR.**	☐	☐	☐	☐	☐	☐
3. O local onde eu trabalho (Ex.: clínica, hospital) geralmente encaminha todos os pacientes elegíveis para RC.*<br>*3. The place where I work (e.g., clinic, hospital) generally refers all eligible patients to CR.**	☐	☐	☐	☐	☐	☐
4. Convênios médicos são um desincentivo para o encaminhamento para RC.<br>*4. Health insurance plans are a disincentive for referring patients to CR.*	☐	☐	☐	☐	☐	☐
5. O acompanhamento do paciente, incluindo seu encaminhamento para RC, é feito por outro profissional da saúde.<br>*5. The patient's follow-up, including their referral to CR, is handled by another healthcare professional.*	☐	☐	☐	☐	☐	☐
6. Eu geralmente tenho a intenção de encaminhar meus pacientes para RC.*<br>6. I usually intend to refer my patients to CR.*	☐	☐	☐	☐	☐	☐
7. Não conheço os programas de RC da minha região.<br>*7. I am not familiar with the CR programs in my region.*	☐	☐	☐	☐	☐	☐
8. Não conheço os programas de RC fora da minha região.<br>*8. I am not familiar with the CR programs outside my region.*	☐	☐	☐	☐	☐	☐

The Portuguese Version of the Provider Attitudes Toward Cardiac Rehabilitation & Referral Scale - Revised
***Atitudes Médicas em Relação à Reabilitação Cardíaca e Encaminhamento para os Programas* (PACRR-P-R)**

9. Onde trabalho, não há um protocolo padrão de encaminhamento para RC, o que torna mais difícil encaminhar os pacientes.<br>*9. Where I work, there is no standard referral protocol for CR, which makes it more difficult to refer patients.*	☐	☐	☐	☐	☐	☐
10. Onde trabalho, outro professional de saúde encaminha o paciente ao programa de RC no meu lugar.<br>*10. Where I work, another healthcare professional refers the patient to the CR program on my behalf.*	☐	☐	☐	☐	☐	☐
11. É trabalhoso fazer o encaminhamento de um paciente para RC.<br>*11. Referring a patient to CR is a cumbersome process.*	☐	☐	☐	☐	☐	☐
12. Eu mesmo(a) prefiro reabilitar o meu paciente.<br>*12. I prefer to rehabilitate my patient myself.*	☐	☐	☐	☐	☐	☐
13. Onde trabalho tenho materiais educacionais para o paciente que são suficientes para promover mudanças de comportamento.<br>*13. Where I work, we have sufficient educational materials for patients to promote behavior change.*	☐	☐	☐	☐	☐	☐
14. Eu mesmo(a) posso prescrever exercícios físicos para o paciente.<br>14. I can prescribe physical exercise to my patients myself.	☐	☐	☐	☐	☐	☐
15. Não quero gastar meu tempo com encaminhamentos, pois meus pacientes não estão motivados a participar da RC ou a se exercitar.<br>*15. I do not want to spend my time on referrals because my patients are not motivated to participate in CR or exercise.*	☐	☐	☐	☐	☐	☐
16. Eu não acredito nos benefícios da RC para os meus pacientes.<br>16. I do not believe in the benefits of CR for my patients.	☐	☐	☐	☐	☐	☐
17. O programa de RC disponível é de baixa qualidade.<br>17. The available CR program is of low quality.	☐	☐	☐	☐	☐	☐
18. Já tive uma experiência negativa com um programa de RC (por exemplo, paciente relatou não ter gostado do programa, não recebeu relatório de evolução do paciente, paciente teve eventos adversos graves, programa mudou os medicamentos do paciente).	☐	☐	☐	☐	☐	☐

18. I have had a negative experience with a CR program (e.g., the patient reported not liking the program, no patient progress report was received, the patient had serious adverse events, the program changed the patient's medication).						
19. Os pacientes têm muitas barreiras para frequentar a RC (por exemplo, não pode pagar as sessões e/ ou o transporte, distância, conflitos com horários das sessões), então não adianta os encaminhar. *19. Patients face many barriers to attending CR (e.g., cannot afford the sessions and/or transportation, distance, scheduling conflicts with session times), so there is no point in referring them.*	☐	☐	☐	☐	☐	☐
20. Frequentemente tenho preocupações sobre segurança ou risco em RC relacionados ao estado clínico do meu paciente. *20. I frequently have concerns about safety or risks in CR related to my patient's clinical condition.*	☐	☐	☐	☐	☐	☐
21. Quais são os fatores mais importantes que afetam seu encaminhamento de pacientes para RC? *21. What are the most important factors that affect your referral of patients to CR?*						

*Itens com escore reverso (*Reverse scored items*).

RC = reabilitação cardíaca (*cardiac rehabilitation*). N/A = não se aplica (*not applicable*).

Options are: Strongly agree, Agree, Neutral, Disagree, Strongly disagree, Not applicable
